# Geographic and seasonal variations in hip fracture incidence in Sweden: a nationwide population-based study

**DOI:** 10.1007/s11657-025-01652-y

**Published:** 2026-01-03

**Authors:** Katarina Greve, Stina Ek, Erzsébet Bartha, Maria Sääf, Margareta Hedström, Karin Modig

**Affiliations:** 1 Department of Clinical Science, Intervention and Technology (CLINTEC), Karolinska Institutet, Stockholm, Sweden; 2 Function Perioperative Medicine and Intensive Care (PMI), Karolinska University Hospital, Stockholm, Sweden; 3 Institute of Environmental Medicine, Unit of Epidemiology, Karolinska Institutet, Stockholm, Sweden; 4 Department of Molecular Medicine and Surgery, Karolinska Institutet, Stockholm, Sweden; 5 Trauma and Reparative Medicine Theme (TRM), Karolinska University Hospital, Stockholm, Sweden

**Keywords:** Hip fractures, Epidemiology, Seasonal differences

## Abstract

***Summary*:**

Rationale: To investigate geographic and seasonal differences in the occurrence of hip fracture within Sweden in the years 2020–2022. Main Results: There were differences in hip fracture incidence rates depending on geographic location as well as sex and season. Significance: Unexplained differences in hip fracture occurrence remain, warranting further investigation.

**Purpose:**

To investigate geographic differences in hip fracture incidence in Sweden and to explore potential factors underlying this variation.

**Methods:**

The populations of people ≥ 60 years of each Swedish county were identified using the Total Population Register. First hip fractures occurring between 2020 and 2022 were identified in the National Patient Register. Sex-, and County specific as well as seasonal hip fracture incidence rates were calculated. Stratified Poisson regression was used to compare incidence in the first quarter of the year with that in the remaining quarters. Additionally, sex stratified adjusted Poisson regression was performed to compare the incidence in Stockholm with other counties.

**Results:**

2,879,444 individuals were included in the study. Hip fracture incidence rates varied across Swedish counties, with the age standardized rate per 10,000 person-years ranging from 54 (Kalmar) to 67 (Västernorrland) in women and from 35 (Södermanland) to 52 (Norrbotten) in men. Incidence tended to be lower during the summer months, except among women 60–79 years in southern Sweden. Seasonal variations were most pronounced among men 60–79 years in northern Sweden. Geographic differences persisted after adjusting for age, income level, care dependency, hospital frailty risk score, place of birth, and usage of fall-risk increasing drugs.

**Conclusion:**

Geographic disparities in first hip fracture incidence were observed among both women and men in Sweden, with the highest rates in the northernmost counties. These differences could not be fully explained by uneven distribution of the risk factors examined in this study. Additionally, sex differences were noted in the counties with the highest and lowest incidence rates. Seasonal variation in hip fracture incidence was evident.

**Supplementary Information:**

The online version contains supplementary material available at 10.1007/s11657-025-01652-y.

## Introduction

Hip fracture is the most severe fragility fracture, affecting around 14,000 to 16,000 people per year in Sweden [1]. Some of the known risk factors for sustaining a hip fracture are advanced age [2, 3], osteoporosis [2], smoking [4], alcohol use [4], physical inactivity [5], low BMI [4], co-existing diseases such as diabetes mellitus [6], and medications that can affect either bone mineral density or risk of falling [7]. Women are more prone to hip fracture than men [3] due to several factors – higher prevalence of osteoporosis [2], differences in bone metabolism [8] and in bone structure [9], as well as a higher prevalence of falls [10] compared with men.

Socioeconomic factors have also been associated with hip fracture risk [11, 12], likely due to the association between socioeconomic status and many of the risk factors mentioned above, but also due to differences in living environment and activities between social groups. Additionally, ethnicity associated genetic and biomechanical factors are also likely to be of importance [3], with the Scandinavian countries having among the highest hip fracture incidence in the world [2]. Protective factors include pharmacologic osteoporosis treatment [13] and total hip arthroplasty [14], with the latter making it impossible for an individual to sustain a “classic” hip fracture on the operated hip, although the risk of periprosthetic fractures remains.

Differences in hip fracture incidence between geographic regions have been reported from many other countries, for example Finland [15], Norway [16], the U.K. [17], Germany [18] and the U.S. [19]. In Sweden, higher incidence has been reported in the north of the country [20–22]. Sweden is an elongated country, stretching between latitude 55°N in the south and 69°N in the north, with the latitude of the arctic circle at approximately 66°N. A previously suggested explanation to increased hip fracture rates in northern Sweden is that people living in higher latitudes may have vitamin D deficiency related to lower exposure to sunlight in the winter, leading to lower bone mineral density [20, 21]. However, other studies suggest that the association between less sunlight and higher incidence of hip fractures may be due to other factors, since the peak yearly incidence of hip fracture tended to precede the expected seasonal nadir of bone mineral density [23, 24]. Furthermore, in contrast with Sweden, a higher hip fracture incidence in the north has not been confirmed in studies from Finland and Norway, two of Sweden’s closest neighboring countries, which both extend even farther north [15, 16].

Seasonal variations in hip fracture incidence have been confirmed in studies from across the world, with a higher incidence in winter compared with summer [21, 23–27]. Additionally, studies from Sweden [21] and Norway [25] have reported a statistical interaction between geographic region of residence and season.

Potential explanations for regional differences in hip fracture incidence in Sweden other than sun exposure include differences in climate, with more snow and ice in the north in the winter months, which could lead to slippery walking conditions outdoors, more time spent indoors and less physical activity. There could also be regional differences in the distribution of numerous known and unknown other factors associated with the risk of sustaining a hip fracture.

Given this background, an updated understanding of how hip fracture incidence varies across counties in Sweden is of great interest. Such insight can not only support health care planning, but also contribute to identifying potential underlying risk factors for hip fractures and, by extension, inform preventive strategies.

### Purpose

This study aimed to investigate and describe the county- and sex-specific incidence rate of first hip fractures in Sweden in the period 2020–2022, both overall and after age standardization. Additionally, to explore whether regional differences in hip fracture incidence could be explained by factors such as seasonal variation or differences in population characteristics.

## Methods

### Study design, setting and participants

This population-based cohort study included individuals ≥ 60 years living in Sweden between January 1 st, 2020, and December 31 st, 2022. The cohort was open, meaning that individuals who turned 60 during the study period entered the cohort at the start of the calendar year of their 60th birthday and contributed person-time from that point on. Hip fractures were identified by International Statistical Classification of Diseases Version 10 (ICD-10) codes S720-722 occurring as primary causes for hospitalization from the National Patient Register (NPR). Individuals who had a previous diagnosis code, and thus hospitalization, for hip fracture in NPR in the last 20 years before the study period were excluded from the study population.

### Outcome variables

- Crude and age standardized county- and sex-specific incidence rates of first hip fracture, reported as number of hip fractures per 10,000 person-years, for the whole study period as well as with the year divided into quarters.
- Incidence rate ratios (IRRs) of first hip fracture of the first quarter of the year compared with quarters 2, 3 and 4.
- Adjusted IRRs of first hip fracture of each county compared with a reference county (Stockholm).

Exposure variable: County of residence. Sweden is divided into 21 counties that consist of several municipalities (except for Gotland, an island in the Baltic Sea that is both a municipality and a county). A map of Sweden and its counties can be found in the appendix (SI Fig. 1). The population density in 2020 ranged from 2.6 people/km^2^ in Norrbotten, the northernmost county, to 367.2 people/km^2^ in Stockholm county [28] and the he average population density of Sweden, 25.5 people/km^2^, was lower than the average population density of Europe, which was 34 people/km^2^ [29]. Average sunshine duration 2008–2020 varied over the country with highest levels by the coasts and lowest levels in the mountainous area in northwestern Sweden and inland southern Sweden. In the very north, there are extreme differences over the year with midnight sun in the summers and no sunshine at all during parts of the winter [30]. This study did not consider where the hip fractures were sustained or treated, but only where the person suffering the fracture was registered. County of residence was defined as the registered county of residence in December immediately preceding each study year, e.g. December 2019, 2020, and 2021.

**Fig. 1:**
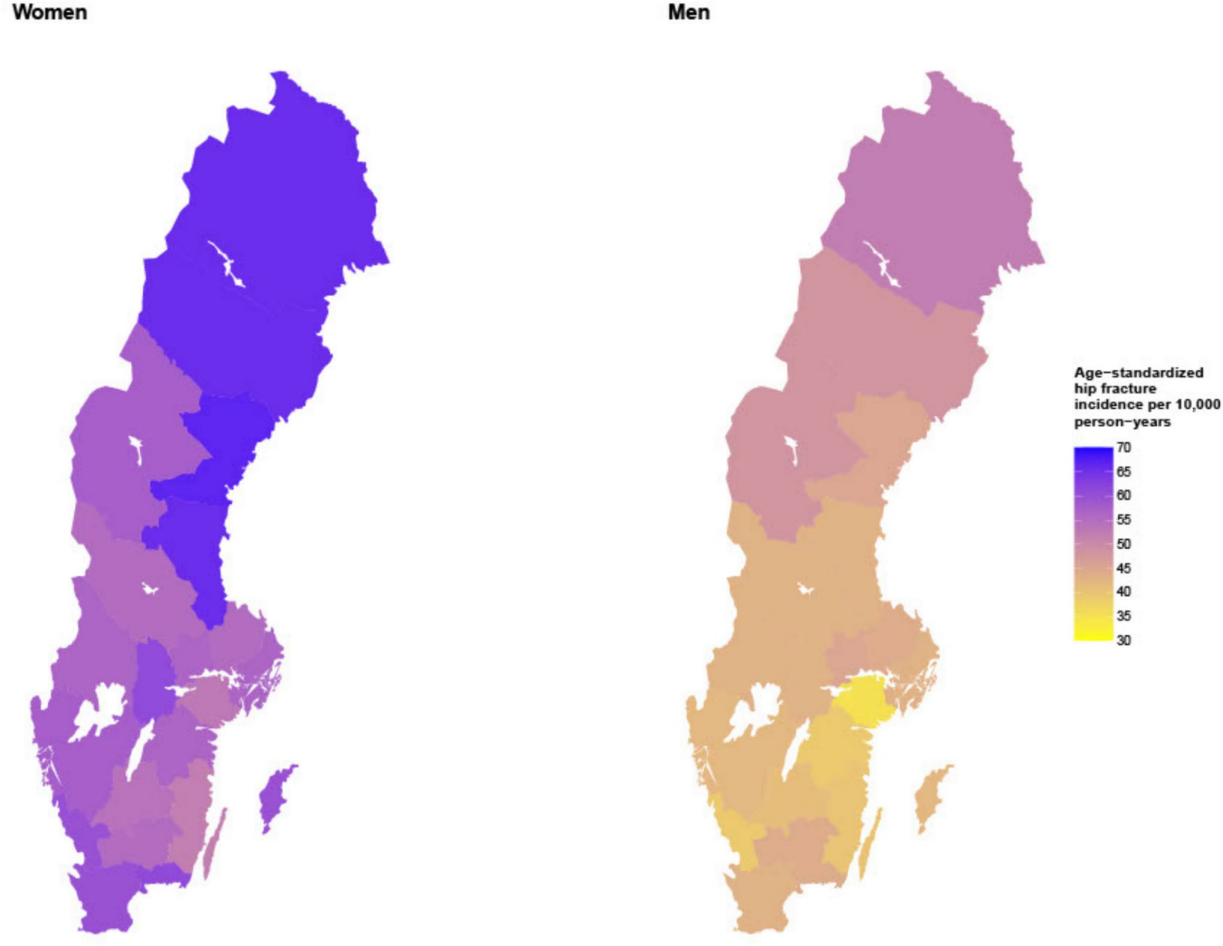
Sex-and county specific incidence rates of hip fracture in Sweden, age standardized

### Data sources

This study used several different administrative registers with mandatory participation for the whole Swedish population. TPR [31] collects information of all the residents of Sweden and includes information such as residence, age, sex, and marital status. It tracks changes in the population related to births, death and migration, enabling generation of official statistics and epidemiological research. NPR [32–34] holds information on in-patient and specialist out-patient care in Sweden, including ICD-10 codes registered in conjunction with doctors’ visits or discharge from hospital stays. The completeness for hip fracture diagnoses in NPR is considered high since patients with acute hip fractures virtually always are hospitalized. The Longitudinal Integration Database for Health Insurances and Labour Market Studies (LISA) holds information on income, education and unemployment [35]. The National Register of Care and Social Services for the Elderly and Persons with Impairments (SSR) holds data on publicly funded elder care in Sweden, as reported by the municipalities. This type of elder care constitutes a vast majority of the professional elder care in Sweden [36]. The National Prescribed Drug Register PDR [34] holds information on all drugs dispensed from a pharmacy in Sweden. Data linkage across different registers was enabled by the Personal Identification Number (PIN) assigned to every person residing legally in Sweden for more than one year. The PINs were pseudonymized before data were accessible to the researchers to protect the identity of the study subjects.

### Risk factors for hip fracture

For the regression analyses comparing counties, several risk factors for hip fracture that could potentially explain regional differences were identified a priori. All analyses were stratified by sex and adjusted for age. Co-morbidities were defined as Hospital Frailty Risk Score (HFRS) [37], a score that is constructed from weighted diagnostic codes found in NPR, in this study looking back two years. HFRS was categorized into < 5, 5–15 and > 15. Socioeconomic status was defined from level of disposable income per consumption unit from LISA [35]. This measure considers the income of the whole household, which prevents individuals with low income living with high income spouses from being classified as deprived and vice versa. Income was then divided into overall study population quintiles. Care dependency was defined from home care utilization and living situation as reported in SSR and divided into four categories with increasing levels of care dependency: no municipal care; living in a private residence with municipal instrumental care (assistance with “practical” tasks such as home cleaning and grocery shopping); living in a private residence with municipal personal care (assistance with tasks such as personal hygiene and eating); and, finally, living either in a care home or in a private residence with ≥ 744 h per month of municipal home care, corresponding to care around the clock. Care dependency is recorded monthly in SSR and for this study, the highest level of care dependency looking back one year was used. Those missing from SSR were classified as receiving no municipal care. Place of birth was categorized into Sweden, Nordic country except Sweden and other. Data on disposed prescriptions of fall-risk increasing drugs (FRIDs) [38] were collected from PDR, looking back one year, and categorized into 0, 1, 2 or 3 + classes of FRIDs. A list of the drug classes considered FRIDs can be found in the appendix (SI Table 1). Use of osteoporotic medications was not included in the analyses due to incomplete individual level data on their utilization [39].

### Statistical methods

County- and sex-specific incidence rates were calculated as number of hip fractures divided by person-years at risk in each county, separate for women and men. The data was split at each calendar year to enable individuals to move between counties and to enter the cohort after the start of the study period. Individuals were censored at hip fracture, death, or emigration. Heatmaps of Sweden were constructed to visualize the regional differences in incidence. Secondly, sex and county-specific crude and age-standardized incidence were calculated for each quarter of the year (Q1: January to March, Q2: April to June, Q3: July to September, Q4: October to December) to elucidate whether there were seasonal differences in hip fracture incidence, and whether these varied based on county of residence. This analysis was repeated after dividing the population into two age groups, ≤ 79 years and ≥ 80 years, and the country into two parts based roughly on the Köppen-Geiger Climate Classification of Sweden: north with subarctic climate and south with humid continental climate [40] (SI Table 2). Stratified Poisson regression was used to compare quarter 1 with quarters 2,3 and 4. A sensitivity analysis was performed, including only people who had lived in the same county for at least 20 years, ensuring a substantial exposure to county of residence. For this analysis, domestic migration to another county within Sweden also constituted a censoring event. Age-standardization was performed by indirect standardization using the total Swedish population ≥ 60 years at dec 31 st 2020 in 5-year age-groups as the standard population. This standard can be found at Statistics Sweden [41]. Poisson regression was used to estimate adjusted IRRs for each region compared with every other region, adjusted for age in 5-year age-groups, level of disposable income, care dependency, HFRS, place of birth, and FRIDs. SAS was used for data management and statistical analyses were performed using STATA (StataCorp. 2021. 136 Stata Statistical Software: Release 17. College Station, TX: StataCorp LLC). Heatmaps were generated using RStudio (Posit team [2024]. RStudio: Integrated Development Environment for R. Posit Software, PBC, Boston, MA.http://www.posit.co/).

## Results

In total, 2,879,444 individuals were included in the study of which 1,497,293 were women and 1,382,151 men. The average yearly number of first hip fractures during the study period was 8,853 for women and 4,822 for men. Further descriptive characteristics of the study population can be found in SI Tables 3 and 4.

### Regional incidence of first hip fractures

Clear regional differences in hip fracture incidence were found. The crude hip fracture incidence ranged between 59 per 10,000 person-years in Uppsala, in the southern-middle part of Sweden and 73 per 10,000 person-years in Västernorrland (in the northeast) for women; and between 32 per 10,000 person-years in Södermanland (in the southern-middle part of Sweden) and 52 per 10,000 person-years in Norrbotten (in the north) for men (Tables 1 and 2). A map of Sweden and its counties can be found in the appendix (SI Fig. 1). After age-standardization, the lowest incidence for women was found in Kalmar, in the southeast, while the highest remained in Västernorrland. For men, the county with the lowest and highest incidence of hip fracture did not change after age-standardization. For a visual description of the regional age standardized incidence, see Fig. 1. Age standardization did not eliminate the regional differences in first hip fracture incidence.

**Table 1:** Number of first hip fractures, crude and age-standardized incidence rate and incidence rate ratios, all counties in Sweden, women ≥ 60 years

County	Number of hip fractures	Crude incidence of hip fractures per 10,000 person-years	Age standardized incidence of hip fractures per 10,000 person-years	Adjusted* incidence rate ratio with 95% confidence intervals for each region compared with Stockholm
Blekinge	518	71	62	1.05 (0.96–1.15)
Dalarna	851	63	57	**0.92 (0.85–0.99)**
Gotland	197	64	61	1.01 (0.88–1.17)
Gävleborg	944	71	66	**1.09 (1.02–1.17)**
Halland	981	68	61	0.99 (0.93–1.07)
Jämtland	363	62	59	0.97 (0.87–1.08)
Jönköping	958	65	56	**0.91 (0.85–0.98)**
Kalmar	716	60	54	**0.86 (0.79–0.93)**
Kronoberg	530	65	57	0.92 (0.84–1)
Norrbotten	831	71	66	1.06 (0.98–1.14)
Skåne	3560	66	61	**1.05 (1–1.09)**
Stockholm	4822	60	58	Ref
Södermanland	788	60	55	0.95 (0.88–1.02)
Uppsala	849	59	57	0.96 (0.89–1.03)
Värmland	861	65	58	**0.92 (0.85–0.99)**
Västerbotten	802	71	66	1.02 (0.94–1.1)
Västernorrland	835	73	67	**1.09 (1.01–1.17)**
Västmanland	770	66	58	**0.91 (0.85–0.99)**
Västra Götaland	4312	64	59	1.01 (0.97–1.05)
Örebro	860	67	62	1.04 (0.96–1.11)
Östergötland	1212	64	58	**0.92 (0.86–0.98)**
Sweden	26 560	64	59	

*Adjusted for: age, Hospital Frailty Risk Score, socioeconomic status, fall-risk increasing drugs, country of birth and care dependency

**Table 2:** Number of hip fractures, crude and age-standardized incidence rate and incidence rate ratios, all counties in Sweden, men ≥ 60 years

County	Number of hip fractures	Crude incidence of hip fractures per 10,000 person-years	Age standardized incidence of hip fractures per 10,000 person-years	Adjusted* incidence rate ratio with 95% confidence intervals for each region compared with Stockholm
Blekinge	286	42	44	0.97 (0.85–1.09)
Dalarna	495	38	43	**0.89 (0.8–0.98)**
Gotland	110	39	42	0.92 (0.76–1.11)
Gävleborg	494	39	43	0.94 (0.85–1.04)
Halland	479	36	39	**0.84 (0.76–0.93)**
Jämtland	243	43	48	1.03 (0.9–1.17)
Jönköping	520	38	41	**0.87 (0.79–0.96)**
Kalmar	419	38	40	**0.84 (0.76–0.93)**
Kronoberg	322	41	44	0.96 (0.85–1.08)
Norrbotten	509	46	52	1.07 (0.97–1.17)
Skåne	1942	40	43	0.99 (0.94–1.05)
Stockholm	2540	36	43	Ref
Södermanland	391	32	35	**0.78 (0.71–0.88)**
Uppsala	505	38	44	0.96 (0.88–1.06)
Värmland	490	40	43	**0.9 (0.82–0.99)**
Västerbotten	457	43	48	0.96 (0.87–1.06)
Västernorrland	430	40	45	0.94 (0.85–1.04)
Västmanland	456	42	45	0.92 (0.83–1.01)
Västra Götaland	2305	37	42	0.95 (0.9–1.01)
Örebro	452	39	43	0.95 (0.86–1.05)
Östergötland	620	36	39	**0.81 (0.75–0.89)**
Sweden	14 465	38	43	

*Adjusted for: age, Hospital Frailty Risk Score, socioeconomic status, fall-risk increasing drugs, country of birth and care dependency

### Seasonal differences

When dividing the calendar year into quarters (Q1-Q4), most counties had the highest age-standardized hip fracture incidence in either Q1 or Q4, i.e. in the winter months. Only four counties (Kronoberg, Gotland, Halland, Örebro) among women and one county (Gotland) among men deviated from this pattern (SI Table 5).

### Regression analyses

Poisson regression comparing the yearly quarters stratified by sex, age-group (60–79 and ≥ 80) and part of the country (north and south) revealed that women aged 60–79 years in the north had statistically significantly lower incidence of first hip fracture in Q2-3 compared with Q1. Women aged 60–79 years in the south had higher incidence in Q3 and Q4 compared with Q1. Among women aged ≥ 80 years, those living in the south had lower incidence in Q2 compared with Q1. There were no statistically significant differences in hip fracture incidence depending on season for women aged ≥ 80 years in the north (Table 3).

**Table 3:** Incidence rate ratios and 95% confidence intervals estimated with Poisson regression, quarters of the calendar year (2020–2022), women

	Q1 vs all other Qs, ages 60–79				Q1 vs all other Qs, ages ≥ 80			
Region	Q1	Q2	Q3	Q4	Q1	Q2	Q3	Q4
Southern Sweden	Ref	0.96 (0.9–1.03)	**1.07 (1–1.15)**	**1.11 (1.03–1.18)**	Ref	**0.94 (0.9–0.98)**	0.97 (0.93–1.02)	1.04 (0.99–1.09)
Number of fractures, southern Sweden	1640	1582	1779	1821	3919	3628	3707	3858
Northern Sweden	Ref	**0.81 (0.7–0.94)**	**0.84 (0.73–0.97)**	1.03 (0.89–1.18)	Ref	0.92 (0.84–1.02)	1.01 (0.92–1.12)	1.06 (0.96–1.17)
Number of fractures, northern Sweden	412	336	351	425	798	725	783	796

Boldface numbers indicate statistical significance

For men, those aged 60–79 years living in the south had statistically significantly lower hip fracture incidence in Q2 and Q3 compared with Q1. For the men aged 60–79 years living in the north, Q2, Q3 and Q4 all had lower incidence than Q1. For the men aged ≥ 80 years in southern Sweden, Q2 and Q3 had statistically significantly lower incidence than Q1. For the men aged ≥ 80 years in the north, Q2 had lower incidence than Q1 (Table 4).

**Table 4:** Incidence rate ratios and 95% confidence intervals estimated with Poisson regression, quarters of the calendar year (2020–2022), men

	Incidence rate ratios and 95% confidence intervals Q1 vs all other Qs, ages 60–79				Incidence rate ratios and 95% confidence intervals Q1 vs all other Qs, ages ≥ 80			
Region	Q1	Q2	Q3	Q4	Q1	Q2	Q3	Q4
Southern Sweden	Ref	**0.82 (0.76–0.89)**	**0.87 (0.81–0.95)**	0.99 (0.91–1.07)	Ref	**0.86 (0.8–0.92)**	**0.93 (0.87–0.99)**	1.04 (0.98–1.11)
Number of fractures, southern Sweden	1268	1043	1119	1254	1923	1621	1723	1886
Northern Sweden	Ref	**0.62 (0.52–0.73)**	**0.72 (0.61–0.84)**	**0.77 (0.66–0.91)**	Ref	**0.81 (0.7–0.93)**	0.88 (0.77–1.01)	0.93 (0.81–1.07)
Number of fractures, northern Sweden	349	216	253	270	438	347	374	381

Boldface numbers indicate statistical significance

Further regression analyses compared individual counties while adjusting for age, co-morbidities, socioeconomic status, FRIDs, birth country and care dependency. Sex specific IRRs of each county compared with Stockholm, the most populous county in Sweden, are presented in Table 1 and Table 2, and heat maps depicting these results can be found in the supplementary information (SI Fig. 2). While some differences persisted after adjustment, some differences became non-significant when accounting for the mentioned covariates. Moreover, for some counties there were no differences in the age standardized IR, while differences appeared after accounting for the covariates, e.g. Västmanland and Östergötland in women, and Dalarna and Värmland in men.

Among women, the county with the highest age standardized incidence rate, Västernorrland, had an adjusted IRR of 1.09 (95% CI 1.01–1.17), i.e. still higher incidence than Stockholm, but only barely statistically significant. The county with the lowest age standardized incidence, Kalmar, also had lower adjusted incidence than Stockholm, IRR 0.86 (95% CI 0.79–0.93), (Table 1).

Among men, Norrbotten, the county with highest age standardized incidence, had an IRR of 1.07 (95% 0.97–1.17), i.e. not statistically significantly different from Stockholm when adjusting for potential explanatory factors. In fact, after adjustment, none of the other counties had significantly higher incidence than Stockholm and seven counties had a lower incidence. Södermanland, the county with the lowest age standardized incidence among men, had IRR 0.78 (95% CI 0.71–0.88) compared with Stockholm (Table 2).

### Sensitivity analysis of those having lived in the same county for 20 years

Sex-and County specific crude and age standardized first hip fracture incidence is displayed in SI tables 6 and 7. The age standardized incidence for those who had lived in the same county at least 20 years tended to be slightly lower than the incidence in the whole population studied.

## Discussion

### Key results

This study confirms the presence of regional differences in first hip fracture incidence in Sweden. These differences were not fully explained by variations in age or other key risk factors adjusted for in regression analyses, although some of the differences between counties either disappeared or were amplified. Notably, there were distinct sex-specific patterns in regional hip fracture incidence with different counties exhibiting the highest and lowest age-standardized incidence rates depending on sex (Tables 1 and 2, Fig. 1).

Consistent with previous research [20, 21], the northernmost part of the Sweden had the highest age standardized hip fracture incidences for both sexes. However, the lowest incidence rates were not observed in the very southernmost counties (Fig. 1). This may, in part, be attributed to urbanization effects – previous research has demonstrated higher incidence of hip fractures in urban areas compared with rural ones [3, 42, 43], with one suggested mechanism being higher physical activity levels among rural residents [42]. As southern Sweden has a higher population density than northern Sweden, a common measure of urbanization [43], this factor could contribute to the observed geographical patterns.

Regional differences in hip fracture incidence have been reported from Norway, with lower incidence in the west than the east and highest incidence in the capital Oslo, located in the southeast [16], perhaps due to factors associated with urbanization. However, recent research report that the incidence rate of hip fracture has been declining in Oslo, and that the incidence rate in Oslo is now below the Norwegian national average. The authors discuss that increased use of anti-osteoporotic drugs, a healthier older population and/or a higher proportion of immigrants could be part of the explanation [44]. In Finland, there was also an east–west gradient, but the lowest incidence was in the east. The Finnish study reported a convergence of hip fracture incidence between geographic regions over time, hypothesizing that the convergence was due to improved living conditions and longevity in eastern Finland, factors that in turn are associated with increased hip fracture incidence [15].

We conducted a sensitivity analysis restricted to individuals who had lived in the same county for 20 years (the majority of the main study population), to ensure a long geographical exposure of a given county. The results were similar as the main findings, (SI tables 6 and 7), suggesting that living in the same area for a long time did not accentuate the regional differences.

### Seasonal differences

When investigating seasonal variations in hip fracture incidence, we found that most counties had highest incidence in the winter months. Seasonal differences appeared to be more pronounced among men. This is consistent with findings from a previous Swedish study [21] and a study from Norway [25]. Our findings may be explained by an increased tendency for younger individuals to engage in outdoors activities, making them more vulnerable to icy conditions in the winter compared to older individuals. Regarding differences in seasonal variations between women and men, it can be hypothesized that men may be more likely to perform outdoor chores, such as shoveling snow, during the winter. This hypothesis is supported by a study on falls in older adults in Boston, which reported more outdoor falls and on snowy and icy surfaces among men than women [45], as well as a recent Swedish study, which found that the average person sustaining a hip fracture due to slipping on ice and snow was more likely to be male and slightly younger, compared with the average patient with hip fracture [46]. However, the location and circumstances of the events leading to hip fractures are not available from national registers. Of note in this present study is that the highest hip fracture incidence tended to be in quarter 4 rather than quarter 1, where one would expect the levels of vitamin D to be at their lowest. This is in line with research from the U.K. [23, 24] and contradicts the theory of vitamin D depletion being an important factor in seasonal variations in hip fracture incidence in Sweden [20, 21].

### Risk factors for hip fracture

While we were able to adjust the regression analyses for age, co-morbidities, socioeconomic status, FRIDs, birth country and care dependency, several other risk factors for hip fracture that could potentially differ between counties were not accounted for. Some of them are difficult to assess in this type of research, for example, there is no reliable nationwide individual level data on smoking, BMI, diet, physical activity or alcohol intake in Sweden. Even if part of these factors should be reflected in co-morbidities and socioeconomic status, differences in lifestyle factors could account for some or all the regional differences in hip fracture incidence.

Additionally, there is limited knowledge regarding the utilization of osteoporotic medications. The National Prescribed Drug Register does not capture drugs dispensed outside of pharmacies, such as those administered directly through hospitals (e.g. intravenous bisphosphonates and denosumab), which are increasingly used in Sweden for osteoporosis treatment [39]. Therefore, individual level data on osteoporotic medications were deemed likely to be incomplete and were not included in the regression analyses. Regional differences in osteoporosis medication have been analyzed on an aggregated level in a previous paper by our team [39]. Treatment levels are still low and even if some of the regional differences could be attributed to differences in osteoporosis medication, it is unlikely to explain all of it. Another unaccounted-for protective factor is the prevalence of total hip arthroplasty. In its 2023 annual report, The Swedish Arthroplasty Register (SAR) describes substantial regional differences (from 170 to 264 hip replacements) in the age-standardized incidence of surgeries for elective hip arthroplasty [47]. It does not report on regional differences in the prevalence of hip arthroplasty, but it would be reasonable to assume that regional differences exist. Combining the data from this present study with data from the Swedish Arthroplasty Register could be an interesting avenue for future research.

Frailty was calculated based on diagnoses in specialized care. There may be regional differences in utilization in specialized versus primary care, and therefore the frailty score could be underestimated in some regions, for example in northern Sweden which is more sparsely populated with long distances to hospitals. This would mean that if some rural counties have higher comorbidity burden than what is indicated by the score, adjustment for frailty would not fully account for the difference in comorbidity between the regions and thus a higher incidence rate in rural regions could still be explained by higher comorbidity.

More research is needed to fully understand the regional differences in hip fracture incidence in Sweden. Future studies should focus on identifying modifiable protective factors in counties with the lowest adjusted incidence rates, which could then be implemented in other regions. Regional policies on fall prevention and elder care may also play a role in reducing hip fracture rates.

### Strengths and limitations

A major strength of this study is its population-wide scope, utilizing nationwide data from several high-quality sources. The power is good, even if less populated counties, such as Gotland, have low absolute numbers of hip fracture which entails greater uncertainty when calculating incidence ratios. This limitation has partially been handled by including data from three calendar years, however, the results from the smaller counties should still be interpreted with some caution.

The years investigated in this study were years when society, including health care and elder care, in Sweden and elsewhere were severely affected by the coronavirus disease (COVID-19) pandemic. It is possible that the pandemic and its consequences affected the hip fracture incidence in the Swedish regions in a way that limits the generalizability of our results to other time periods. Official Swedish statistics on the crude nationwide incidence of any fracture of the femur among (ICD 10-code S72) in people aged > 50 years indicate a slight dip in the incidence during the study years [48]. In contrast, a study from Skåne county failed to demonstrate any relevant differences in the incidence of fracture surgery for the knee and hip during the first and early second waves of the pandemic compared with pre-COVID-19-levels [49].

### Interpretation

Geographic disparities in hip fracture incidence persist in Sweden and is present for both women and men. These differences are not accounted for by regional variation in age, frailty, use of fall-risk–increasing drugs, care dependency, socioeconomic status, or country of birth. The highest incidence was observed in northern Sweden, where seasonal variation was also most pronounced. Notably, winter peaks in hip fracture incidence were particularly evident among younger men aged 60–79 years living in the north. These findings suggest that other environmental or structural factors, such as climate, infrastructure or preventive work may contribute to regional and seasonal differences in hip fracture risk.

## Supplementary Information

Below is the link to the electronic supplementary material.

Supplementary file1 (DOCX 189 KB)

## Data Availability

We have stated that data can only be accessed after an ethical review.

## Declarations

### Accessibility of protocol, raw data, and programming code

The individual level data of this study are protected by Swedish law but can be accessed after receiving approval from ethical vetting.
