# Supplementary material for: Geographic and seasonal variations in hip fracture incidence in Sweden: a nationwide population-based study
Source: Arch Osteoporos. 2026 Jan 3;21(1):19. doi: 10.1007/s11657-025-01652-y (PMC12764589; doi:10.1007/s11657-025-01652-y)
Supplement: Supplementary file 1 — Supplementary file1 (DOCX 189 KB) [file 11657_2025_1652_MOESM1_ESM.docx]

**Supplementary Information (SI):**

**
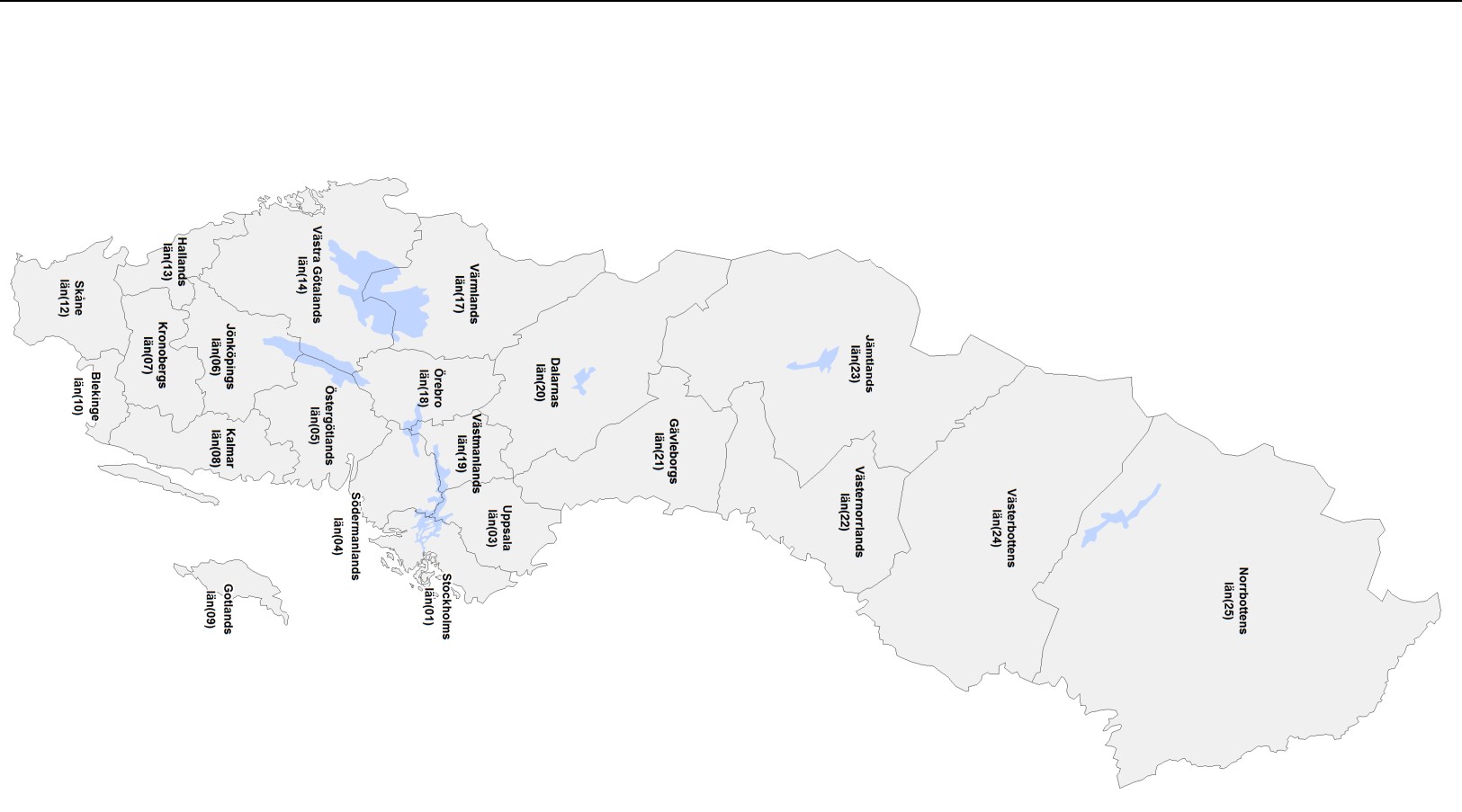
**

**SI Figure 1: map of Sweden and its counties. Source: Statistics Sweden.**

**SI Table 1: List of drug classes considered fall-risk increasing drugs (FRIDs) with Anatomical Therapeutic Chemical Classification System (ATC) codes:**

| C01D: Vasodilators used in cardiac diseases |
| --- |
| C02: Antihypertensives |
| C03: Diuretics |
| C07: Beta blocking agents |
| C08: Calcium channel blockers |
| C09: Agents acting on the renin-angiotensin system |
| G04CA: Alpha-adrenoreceptor antagonists |
| N04B: Dopaminergic agents |
| N02A: Opioids |
| N05A: Antipsychotics (excluding lithium) |
| N05B: Anxiolytics |
| N05C: Hypnotics and sedatives |
| N06A: Antidepressants |

**SI Table 2: Division of Sweden into two parts based on the climate of Sweden according to the Köppen-Geiger climate classification (roughly):**

| South | Stockholm, Uppsala, Södermanland, Östergötland, Jönköping, Kronoberg, Kalmar, Gotland, Blekinge, Skåne, Halland, Västra Götaland, Värmland, Örebro, Västmanland |
| --- | --- |
| North | Dalarna, Gävleborg, Västernorrland, Jämtland, Västerbotten, Norrbotten |

**SI Table 3: Characteristics of the study population, women ≥60 years, with or without hip fracture during the study period:**

| **County** | **Age (mean, SD)^*^** | **Hospital Frailty Risk Score ≥5 (%)^*^** | **Disposable Income ≤2nd quintile (%)^*^** | **Any Municipal Care (%)^*^** | **Place of birth outside the Nordic countries (%)^*^** | **On Any Fall-risk increasing drugs (%)^*^** |
| --- | --- | --- | --- | --- | --- | --- |
| Blekinge | 74 (9) | 5 | 51 | 10 | 7 | 69 |
| Dalarna | 73 (9) | 5 | 49 | 11 | 4 | 66 |
| Gotland | 73 (9) | 6 | 50 | 9 | 3 | 65 |
| Gävleborg | 73 (9) | 5 | 50 | 10 | 4 | 67 |
| Halland | 73 (9) | 6 | 43 | 11 | 8 | 65 |
| Jämtland | 73 (9) | 3 | 50 | 11 | 2 | 67 |
| Jönköping | 73 (9) | 7 | 47 | 12 | 9 | 68 |
| Kalmar | 73 (9) | 6 | 51 | 12 | 6 | 66 |
| Kronoberg | 74 (9) | 5 | 48 | 12 | 10 | 69 |
| Norrbotten | 73 (9) | 6 | 47 | 11 | 2 | 71 |
| Skåne | 73 (9) | 6 | 47 | 10 | 14 | 66 |
| Stockholm | 73 (9) | 8 | 38 | 10 | 17 | 65 |
| Södermanland | 73 (9) | 6 | 47 | 9 | 8 | 67 |
| Uppsala | 73 (9) | 6 | 42 | 10 | 9 | 67 |
| Värmland | 73 (9) | 7 | 50 | 11 | 4 | 69 |
| Västerbotten | 73 (9) | 7 | 46 | 12 | 3 | 71 |
| Västernorrland | 73 (9) | 6 | 48 | 11 | 3 | 70 |
| Västmanland | 73 (9) | 8 | 47 | 13 | 9 | 69 |
| Västra Götaland | 73 (9) | 5 | 46 | 10 | 11 | 66 |
| Örebro | 73 (9) | 6 | 49 | 10 | 7 | 66 |
| Östergötland | 73 (9) | 7 | 48 | 12 | 8 | 67 |
| Sweden | 73 (9) | 6 | 45 | 10 | 10 | 67 |
| ^*^All numbers are rounded to the closest integer | | | | | | |

**SI Table 4: Characteristics of the study population, men ≥60 years, with or without hip fracture during the study period:**

| **County** | **Age (mean, SD)^*^** | **Hospital Frailty Risk Score ≥5 (%)^*^** | **isposable Income ≤2nd quintile (%)^*^** | **Any Municipal Care (%)*** | **Place of birth outside the Nordic countries(%)^*^** | **On Any Fall-risk increasing drugs (%)^*^** |
| --- | --- | --- | --- | --- | --- | --- |
| Blekinge | 73 (8) | 5 | 40 | 7 | 6 | 68 |
| Dalarna | 72 (8) | 6 | 38 | 7 | 3 | 66 |
| Gotland | 72 (8) | 6 | 41 | 6 | 3 | 66 |
| Gävleborg | 72 (8) | 6 | 39 | 7 | 4 | 68 |
| Halland | 72 (8) | 7 | 31 | 7 | 7 | 66 |
| Jämtland | 72 (8) | 4 | 41 | 7 | 2 | 67 |
| Jönköping | 72 (8) | 7 | 35 | 7 | 9 | 67 |
| Kalmar | 73 (8) | 6 | 40 | 7 | 6 | 66 |
| Kronoberg | 73 (8) | 5 | 36 | 7 | 9 | 67 |
| Norrbotten | 72 (8) | 6 | 37 | 7 | 2 | 71 |
| Skåne | 72 (8) | 6 | 36 | 7 | 13 | 66 |
| Stockholm | 72 (8) | 8 | 27 | 6 | 19 | 65 |
| Södermanland | 72 (8) | 6 | 36 | 6 | 8 | 69 |
| Uppsala | 72 (8) | 6 | 31 | 7 | 10 | 67 |
| Värmland | 72 (8) | 7 | 38 | 7 | 4 | 68 |
| Västerbotten | 72 (8) | 7 | 35 | 8 | 3 | 71 |
| Västernorrland | 72 (8) | 7 | 37 | 7 | 3 | 69 |
| Västmanland | 72 (8) | 9 | 35 | 8 | 9 | 68 |
| Västra Götaland | 72 (8) | 5 | 34 | 6 | 11 | 66 |
| Örebro | 72 (8) | 6 | 38 | 7 | 7 | 66 |
| Östergötland | 72 (8) | 7 | 36 | 8 | 8 | 66 |
| Sweden | 72 (8) | 6 | 34 | 7 | 10 | 67 |
| ^*^All numbers are rounded to the closest integer | | | | | | |

**SI Table 5: Region- and sex specific, age standardized first hip fracture incidence for quarters (Q) of the calendar year (2020-2022). The quarter or quarters with the highest incidence for each combination of sex and county is highlighted.**

|  | Age standardized incidence of hip fractures per 10,000 person-years (women) | | | | Age standardized incidence of hip fractures per 10,000 person-years (men) | | | |
| --- | --- | --- | --- | --- | --- | --- | --- | --- |
| County | Q1 | Q2 | Q3 | Q4 | Q1 | Q2 | Q3 | Q4 |
| Blekinge | 62 | 55 | 64 | 68 | 42 | 38 | 47 | 51 |
| Dalarna | 57 | 55 | 56 | 60 | 55 | 33 | 47 | 37 |
| Gotland | 51 | 67 | 60 | 65 | 42 | 37 | 45 | 41 |
| Gävleborg | 69 | 59 | 67 | 70 | 50 | 34 | 42 | 47 |
| Halland | 59 | 56 | 64 | 64 | 38 | 35 | 38 | 44 |
| Jämtland | 61 | 42 | 55 | 78 | 58 | 37 | 41 | 58 |
| Jönköping | 54 | 58 | 53 | 60 | 45 | 39 | 38 | 41 |
| Kalmar | 57 | 47 | 53 | 59 | 46 | 35 | 36 | 44 |
| Kronoberg | 54 | 60 | 58 | 55 | 48 | 41 | 38 | 48 |
| Norrbotten | 70 | 54 | 63 | 79 | 57 | 48 | 47 | 58 |
| Skåne | 57 | 59 | 62 | 66 | 43 | 39 | 44 | 47 |
| Stockholm | 60 | 51 | 58 | 64 | 47 | 37 | 42 | 47 |
| Södermanland | 54 | 55 | 53 | 60 | 33 | 35 | 31 | 41 |
| Uppsala | 57 | 54 | 56 | 59 | 54 | 41 | 37 | 43 |
| Värmland | 55 | 58 | 53 | 65 | 47 | 40 | 35 | 53 |
| Västerbotten | 61 | 69 | 67 | 69 | 56 | 44 | 48 | 44 |
| Västernorrland | 74 | 63 | 66 | 67 | 44 | 46 | 40 | 51 |
| Västmanland | 53 | 59 | 57 | 65 | 45 | 32 | 50 | 53 |
| Västra Götaland | 58 | 55 | 60 | 61 | 43 | 39 | 40 | 46 |
| Örebro | 61 | 63 | 61 | 62 | 44 | 41 | 40 | 49 |
| Östergötland | 60 | 53 | 59 | 60 | 40 | 33 | 41 | 43 |

**
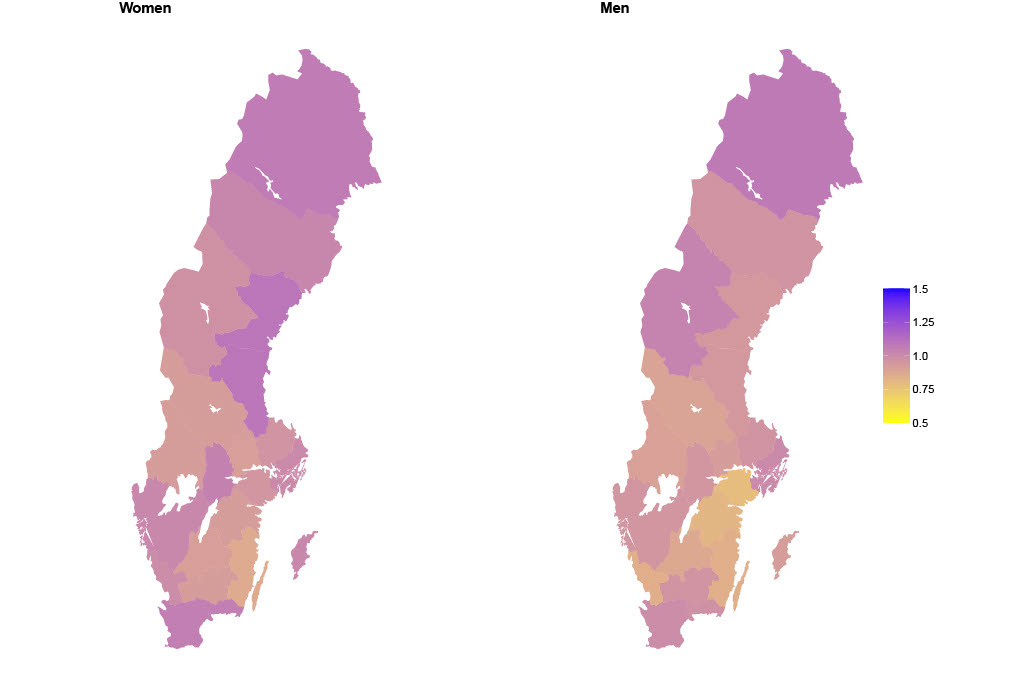
**

**SI Figure 2: Adjusted incidence rate ratios of hip fractures in Sweden among women and men ≥60 years with Stockholm county as the reference for both sexes.**

**SI Table 6: Number of first hip fractures, crude and age-standardized incidence rate, all counties in Sweden, women ≥60 years who had lived in the same county for at least 20 years.**

| County | Number of hip fractures | Crude incidence of hip fractures per 10,000 person-years | Age standardized incidence of hip fractures per 10,000 person-years |
| --- | --- | --- | --- |
| Blekinge | 323 | 76 | 60 |
| Dalarna | 503 | 63 | 52 |
| Gotland | 115 | 68 | 57 |
| Gävleborg | 564 | 71 | 61 |
| Halland | 547 | 70 | 56 |
| Jämtland | 213 | 61 | 53 |
| Jönköping | 572 | 65 | 52 |
| Kalmar | 442 | 65 | 53 |
| Kronoberg | 325 | 68 | 53 |
| Norrbotten | 545 | 75 | 64 |
| Skåne | 2229 | 70 | 58 |
| Stockholm | 2973 | 65 | 55 |
| Södermanland | 443 | 64 | 52 |
| Uppsala | 471 | 62 | 53 |
| Värmland | 517 | 65 | 52 |
| Västerbotten | 508 | 72 | 63 |
| Västernorrland | 515 | 74 | 62 |
| Västmanland | 456 | 68 | 54 |
| Västra Götaland | 2775 | 68 | 57 |
| Örebro | 542 | 72 | 60 |
| Östergötland | 739 | 66 | 54 |
| Sweden | 16 317 | 68 | 56 |

**SI Table 7: Number of first hip fractures, crude and age-standardized incidence rate, all counties in Sweden, men ≥60 years who had lived in the same county for at least 20 years:**

| County | Number of hip fractures | Crude incidence of hip fractures per 10,000 person-years | Age standardized incidence of hip fractures per 10,000 person-years |
| --- | --- | --- | --- |
| Blekinge | 171 | 44 | 43 |
| Dalarna | 266 | 36 | 37 |
| Gotland | 68 | 45 | 45 |
| Gävleborg | 301 | 41 | 43 |
| Halland | 258 | 37 | 36 |
| Jämtland | 143 | 43 | 46 |
| Jönköping | 324 | 41 | 40 |
| Kalmar | 238 | 39 | 38 |
| Kronoberg | 189 | 43 | 42 |
| Norrbotten | 310 | 45 | 48 |
| Skåne | 1175 | 43 | 42 |
| Stockholm | 1568 | 41 | 43 |
| Södermanland | 230 | 37 | 37 |
| Uppsala | 286 | 43 | 44 |
| Värmland | 306 | 43 | 43 |
| Västerbotten | 263 | 41 | 43 |
| Västernorrland | 251 | 39 | 42 |
| Västmanland | 259 | 43 | 42 |
| Västra Götaland | 1441 | 40 | 41 |
| Örebro | 264 | 39 | 41 |
| Östergötland | 355 | 35 | 36 |
| Sweden | 8666 | 41 | 41 |
